# MOVING AND GROOVING: ACTIVITY BENEFITS OF SOCIAL INTEGRATION MAY DEPEND ON RELATIONSHIP QUALITY

**DOI:** 10.1093/geroni/igz038.1170

**Published:** 2019-11-08

**Authors:** Colette J Brown, Karen Rook

**Affiliations:** University of California, Irvine, Irvine, California, United States

## Abstract

New evidence indicates that being more socially integrated increases physical and leisure activities (Fingerman et al., 2019), thus reducing the health risks of being sedentary. Yet socially integrated individuals often have both positive (e.g., supportive) and ambivalent (e.g., supportive and conflictual) relationships. The current study investigated whether the two kinds of relationships exhibit comparable associations with activity levels, and in turn, health. In-person interviews conducted with a national sample of adults ages 65 to 91 (N = 916) assessed participants’ social ties, frequency of vigorous physical activity and leisure activities, and functional health limitations. Hierarchical multiple regression analyses structured to test mediation were conducted. Having more social ties (both positive and ambivalent) was associated with more frequent physical and leisure activity. After adjusting for demographic characteristics, both kinds of social ties were related to health limitations, albeit in opposite directions, with positive ties related to fewer limitations and ambivalent ties related to more limitations. Once physical and leisure activities were included, the association between positive ties and health limitations became nonsignificant, whereas the adverse effect of ambivalent ties remained unchanged. These findings reveal that the health benefits of positive, but not ambivalent, social ties may be explained by physical and leisure activities. This ability to get people up and moving has, until recently, been an overlooked benefit of social integration. Further investigation of the quality of social ties will extend knowledge of the pathways by which they affect health.

